# Molecular mechanism of viscoelastic polymer enhanced oil recovery in nanopores

**DOI:** 10.1098/rsos.180076

**Published:** 2018-06-20

**Authors:** Jing Cun Fan, Feng Chao Wang, Jie Chen, Yin Bo Zhu, De Tang Lu, He Liu, Heng An Wu

**Affiliations:** 1 CAS Key Laboratory of Mechanical Behavior and Design of Materials, Department of Modern Mechanics, University of Science and Technology of China, Hefei, Anhui 230027, People's Republic of China; 2 Zhongtian Technology Submarine Cables Co., Ltd., Nantong, Jiangsu 226010, People's Republic of China; 3 PetroChina Research Institute of Petroleum Exploration & Development, Beijing 100083, People's Republic of China

**Keywords:** polymer flooding, enhanced oil recovery, oil displacement, viscoelasticity, molecular dynamics simulation

## Abstract

Polymer flooding is a promising chemical enhanced oil recovery (EOR) method, which realizes more efficient extraction in porous formations characterized with nanoscale porosity and complicated interfaces. Understanding the molecular mechanism of viscoelastic polymer EOR in nanopores is of great significance for the advancement of oil exploitation. Using molecular dynamics simulations, we investigated the detailed process of a viscoelastic polymer displacing oil at the atomic scale. We found that the interactions between polymer chains and oil provide an additional pulling effect on extracting the residual oil trapped in dead-end nanopores, which plays a key role in increasing the oil displacement efficiency. Our results also demonstrate that the oil displacement ability of polymer can be reinforced with the increasing chain length and viscoelasticity. In particular, a polymer with longer chain length exhibits stronger elastic property, which enhances the foregoing pulling effect. These findings can help to enrich our understanding on the molecular mechanism of polymer enhanced oil recovery and provide guidance for oil extraction engineering.

## 1. Introduction

In order to meet the growing need for petroleum resources, there is an increasing demand to further improve oil recovery factors from mature fields which have already been exploited for years [1,2]. After the reservoir is flooded by water, usually called the secondary oil recovery method, more than 50% of the original oil in place remains unrecovered [3,4]. A critical challenge in conventional oil recovery is the ability to realize more efficient displacement of residual hydrocarbons constrained by a nanoscale porous medium and complicated interfaces. Therefore, enhanced oil recovery (EOR) technologies are urgently needed to displace the large amount of residual oil [5]. Polymer flooding is a promising chemical EOR method through injecting polymer solution (e.g. hydrolysed polyacrylamide) into the oil reservoir [6–8]. Owing to its technical feasibility, commercial advantage and high oil recovery efficiency, polymer flooding has aroused intense interest and been widely used in the world [3,9]. Understanding the displacement mechanism of polymer flooding is of great significance for the advancement of oil exploitation.

Traditional understanding of polymer flooding attributes the improved oil production only to increased macroscale oil recovery efficiency. The injected polymer solution increases the viscosity of the water flood drive fluid and causes disproportionate permeability reduction, so the water–oil mobility ratio is lowered, resulting in a higher volumetric sweep efficiency of the flooded reservoir [10–13]. Nevertheless, recent progress on oil recovery demonstrated that unexpectedly improved oil displacement efficiency of polymer flooding should be attributed to the displacement mechanism at the microscale or nanoscale [14–16]. Saraji *et al*. [17] reported that the average pore radius of several reservoir samples from Bakken formation is within the 5–100 nm range. The characteristics of nanoscale porosity and complicated interfaces of the oil reservoirs result in a considerable amount of petroleum hydrocarbons confined in intricate microscale or nanoscale pores [18–20]. To extract oil in these pores, water flooding is only effective to flush out the petroleum hydrocarbons in the main pore channel [21,22]. A large amount of residual oil is still trapped in dead ends, which are unconnected zones in the pores, leading to a low oil recovery efficiency of water flooding [7,14]. Polymer flooding is reported to have the capacity of extracting oil trapped in the dead ends. Wang *et al*. [14] studied the effectiveness of polymer flooding in displacing residual oil after water flooding and observed a significant reduction of residual oil. The additional oil extraction in dead ends using polymer flooding plays an important role in increasing recovery efficiency.

Flow of non-Newtonian fluid in complex microscopic porous media involves complicated issues [23–25], such as nanoscale confinement, interfacial interactions and viscoelasticity. Several mechanisms have been proposed to explain the improvement of microscopic oil efficiency for polymer flooding. The elastic and viscous properties of the flooding agent play important roles in the residual oil recovery due to the interfacial interactions between hydrocarbons and flooding agent at the atomic scale. The successful oil displacement process requires the flooding agent to provide sufficient ability to overcome oil–wall interactions in silt pores or special confinements. Wang *et al*. [15] found that when a fluid with elastic properties flows over dead ends, normal stresses between oil and flowing fluid are generated in addition to shear stresses. This pulling effect contributes to displacing residual oil in dead ends. When polymer solutions flow through porous media at high velocities, shear thickening occurs due to the elastic property of polymer chains [26]. Clarke *et al*. [27] measured the rheology of polymer solutions in microfluidic networks and observed a clear shear thickening phenomenon which is associated with the onset of elastic turbulence. They also investigated the motion of trapped oil ganglia in a complex three-dimensional structure during polymer flooding using nuclear magnetic resonance diffusion measurements and found that elastic turbulence of a flowing polymer solution is a possible mechanism for enhanced recovery [28]. As for the oil films attached to the rock surface, it is found that the velocity gradient near the wall for elastic fluids is considerably greater than that for Newtonian fluids, causing a stronger force to strip oil films off [29,30].

Those previous efforts help one to understand the oil displacement mechanisms of polymer flooding. However, most of those works focus on oil recovery at a micrometre scale. To our knowledge, there is a lack of understanding of oil recovery by polymer flooding at the atomic scale. The oil displacement process of hydrocarbons trapped in a complex nanoscale environment is still unclear, such as oil droplets confined in the dead end of nanopores. The mechanism of viscoelasticity in polymer flooding needs to be illuminated to achieve a better understanding of oil recovery.

In this presented work, molecular dynamics (MD) simulations were conducted to investigate the microscopic oil recovery mechanisms of viscoelastic polymer EOR. We first studied the dynamic processes of the displacement of an oil droplet originally trapped in a dead end of a nanopore during polymer flooding. Pure water and polymers with different chain lengths were used as flooding agents respectively to evaluate their oil displacing abilities. Moreover, the pulling effect mechanism of polymer flooding was also elucidated on the atomic scale. Finally, based on the oil displacing abilities of polymers with varying chain lengths, we studied the effect of viscoelasticity of polymers on improving microscopic oil displacement efficiency.

## 2. Model and methods

**Figure 1.**
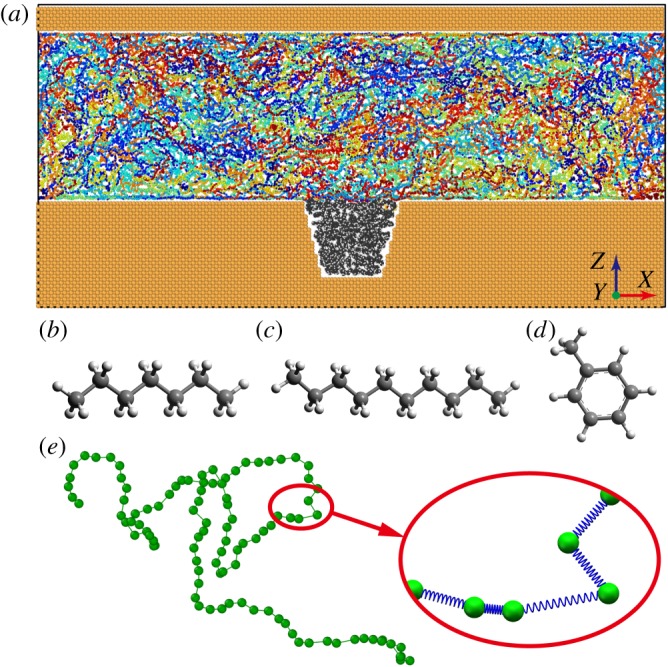
(*a*) Snapshot of the initial structure for MD simulations. The yellow part represents the wall of the pore; the grey part is the oil droplet trapped in the dead end; the colourful chains confined between the walls are polymer molecules. The molecular structures of (*b*) heptane, (*c*) decane and (*d*) toluene are also illustrated. (*e*) The detail of a representative polymer molecule. Inset illustrates the bead-spring model of the polymer chain.

To investigate the microscopic oil displacement mechanism of polymer flooding, an atomic-scale model was set up in MD simulations, as shown in figure 1. An oil droplet was trapped in a wedge-shaped dead end of a pore filled with flooding agent. The surrounding wall of the pore was constructed based on the face-centred cubic (FCC) lattice structure. The multicomponent oil droplet was a ternary mixture of heptane, decane and toluene in a proportion of 3 : 1 : 1. There is other successful research using a similar oil droplet model [31,32]. In this study, we used a simplified polymer chain model proposed by Kremer and Grest (KG) [33]. The beads in the chain represented the monomers of the polymer, and monomers were connected by extensible springs, as shown in figure 1*e*, to consider the viscoelasticity of polymer. The initial configurations of the oil droplet and the polymer flooding agent for the simulations were built via Packmolprogram [34].

The interactions between oil–wall, polymer–wall and oil–polymer were described by the 12-6 Lennard–Jones (LJ) pairwise potential:

2.1 $$U_{\mathrm{LJ}}(r)=4\varepsilon \left[{\left(\frac{\sigma }{r}\right)}^{12}-{\left(\frac{\sigma }{r}\right)}^{6}\right],$$

where *r* is the distance between two atoms, *ε* is the strength of the interaction and *σ* characterizes the distance at which the two atoms are at equilibrium. These interaction parameters are given in table 1. The cut-off distance for the LJ interactions was set to be 12.0 Å.

**Table 1.** LJ parameters for oil–wall, polymer–wall and oil–polymer interactions.

interaction pair *i*, *j*	*ε*_ij_ (kcal mol^−1^)	*σ_ij_* (Å)
polymer–wall	0.1	3.0
oil^a^–wall	0.01	3.0
polymer–oil	0.5	3.0

^a^Oil means every type of atom in heptane, decane and toluene molecules.

The CHARMM force field was used to model the bond, angle, dihedral, van der Waals and electrostatic interactions among the hydrocarbon molecules [35]. The parameters were determined according to a previous study [36]. A switching function was used to ramp the energy and force smoothly to zero between the inner cut-off of 10.0 Å and the outer cut-off of 12.0 Å.

In the KG polymer model, the interactions between non-bonded monomers in the polymer chains were described by a shifted purely repulsive 12-6 LJ potential:

2.2 $$U_{\mathrm{nb}}(r)=\{\begin{array}{ll} 4\varepsilon \left[{\left(\frac{\sigma }{r}\right)}^{12}-{\left(\frac{\sigma }{r}\right)}^{6}\right]+\varepsilon , & r<2^{1/6}\sigma \\ 0 & r>2^{1/6}\sigma \end{array},$$

where *ε* = 0.592 kcal mol^−1^ and *σ* = 2.5 Å. The finite extensible nonlinear elastic (FENE) potential was used to describe the interactions between adjacent bonded monomers:

2.3 $$U_{\mathrm{FENE}}\;(r)=-\frac{kR_{0}^{2}}{2}\ln\left[1-{\left(\frac{r}{R_{0}}\right)}^{2}\right],$$

where *R*_0_ = 1.5*σ* is the spring's maximum extension length and *k* = 30 *ε*/*σ*^2^ is the spring constant. The spring constant is strong enough so that the maximum extension of the bond is always less than 1.2*σ*, which makes bond crossing energetically infeasible [33].

All the MD simulations were carried out in the NVT ensemble with a time step of 1 fs. The size of the simulation box was 342 × 25.2 × 165 Å. Non-periodic boundary conditions were applied in the *Z* direction, and periodic boundary conditions were applied in the *X* and *Y* directions. This quasi-two-dimensional set-up makes it easy to monitor the detail of oil displacement. The width of the pore is 9 nm. The opening width, depth and bottom width of the wedge-shaped dead end are 5 nm, 4 nm and 3 nm, respectively. The temperature was kept constant at 298 K by employing a Nosé–Hoover thermostat. MD simulations in this study were performed using the LAMMPS package [37]. The snapshots of the systems were processed in OVITO software [38].

Polymers with varying chain lengths were used as flooding agents, specifically *N* = 100, 150 and 250, respectively. The total monomer number of different polymers was kept constant at 40 000. Polymer molecules were first modelled as straight chains and randomly placed into a larger box to prepare the starting structures of entangled polymers. The simulation box was then gradually squeezed to the final requested size. These structures were further run at constant volume for an additional 500 ps to achieve equilibrium. Then the entangled polymers and oil droplet were put into the nanopore for the following simulations of oil displacement. In actual working conditions, the flooding agent flow is driven by the pressure gradient. As an equivalent method, here we applied a constant external force along the flow direction (*X* direction) to each polymer monomer. As the scale of the problem is small, the surface effect is prominent, resulting in strong interactions in the system. Hence, a higher pressure gradient is needed to drive the flow. To verify the effectiveness of polymer flooding, water flooding was also performed for comparison using 26 908 water molecules as the flooding fluid. A single point charge/extended (SPC/E) model was used to describe the force field of water molecules [39]. The flooding conditions including temperature and pressure gradient were the same as those in the polymer flooding.

We also carried out another series of equilibrium simulations to analyse the viscoelasticity of polymers and water. In those cases, polymers and water with the same molecule numbers and density of those in the flooding process were randomly embedded in a cubic box with periodic boundary conditions in all directions, and run for up to 500 million steps in equilibration. Similarly, chain lengths of the polymers are *N* = 100, 150 and 250. The autocorrelation function of the total stresses was recorded to calculate the stress relaxation modulus *G*(*t*). Then storage modulus *G*′(*ω*) and loss modulus *G*′′(*ω*) were obtained by Maxwell modes fit to stress relaxation modulus curve of polymers.

## 3. Results and discussion

### 3.1. Polymer flooding

**Figure 2.**
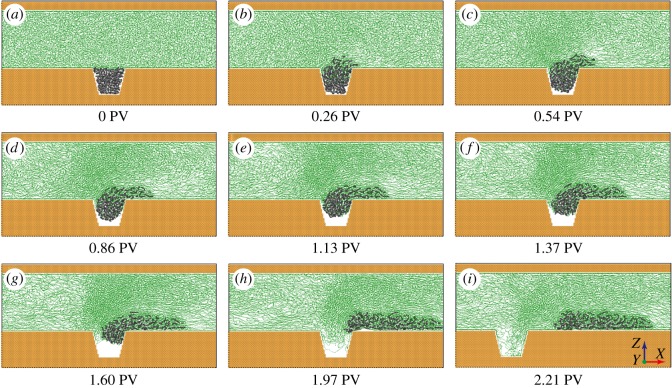
Consecutive snapshots of the polymer flooding process at different injected pore volumes. In this case, polymers with chain length *N *= 150 are flowing along the *X* direction. Owing to the periodic boundary conditions in the *X* direction, we offset the field of view in (*i*) to illustrate the configuration when oil droplet is completely pulled out of the dead end.

Understanding the dynamics of oil displacement is vital in studying the mechanisms of polymer flooding. In our MD simulations, we observed that when the nanopore was flooded by polymers, the whole oil droplet can be pulled out of the dead end. Figure 2 shows a typical flooding process when the oil droplet can be completely displaced. Here, we used the injected pore volume (PV) to measure the accumulated flooding agent injection, which was defined as the ratio of the accumulated number of atoms flowing across the boundary in *X* direction to the total polymer atom number. It should be noted that because the polymer atom number is proportional to the flooding agent volume in our MD model, the definition of PV we used here is equivalent to that in petroleum engineering. With the increase of injected PV, the oil displacement presented in figure 2 can be divided into three stages: (i) after coming into contact with the oil droplet, polymers grab the upper part of the oil droplet and apply pulling force on it, enabling the oil droplet to detach from the bottom surface of the dead end; (ii) as polymers continue to flow, the pulling effect between polymers and the oil droplet overcomes the adhesive interaction between the oil droplet and the solid surface, so the oil droplet is gradually pulled out of the dead end. When the oil droplet is pulled out, it blocks the flowing polymers in the channel and polymers tend to gather near the oil droplet, resulting in the concentration gradient of polymers. Simultaneously, polymers pour into the dead end; (iii) when the oil droplet is completely pulled out of the dead end and then comes to the main pore channel, polymers fill up the dead end, marking the finish of oil displacement.

**Figure 3.**
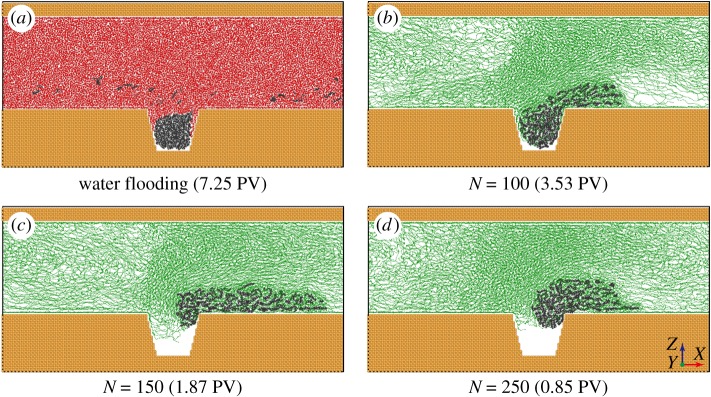
Displacing behaviours of trapped oil by different flooding agents, namely (*a*) water and (*b*–*d*) polymers with varying chain lengths.

Water flooding is widely used in practical oil production applications as a major oil recovery technology. However, the oil recovery efficiency of water flooding is very low compared with that of polymer flooding, even with the same sweep volume. To shed light on the difference between water flooding and polymer flooding at atomic scale, MD simulation of water flooding was performed. The displacing behaviour of oil by water is shown in figure 3*a*. It is clear that even when the injected PV is up to 7.25, only a small amount of hydrocarbon molecules are extracted to the main flow channel, and the majority of the oil droplet is still trapped in the dead end. Note that the hydrocarbon molecules in the left part of the pore channel are due to the periodic boundary conditions in the *X* direction. Unlike polymers that can apply a pulling effect on the oil droplet with entangled chains, the flowing water interacts with the oil droplet only by shearing, resulting in low oil recovery efficiency of water flooding. This shear behaviour only can displace the oil molecules at the water–oil interface, which cannot provide pulling interaction to overcome the oil–wall interaction in nanoscale confinement. This comparison between water flooding and polymer flooding indicates that polymer flooding is indeed able to immensely improve oil displacement efficiency, which is consistent with the experimental observations [14].

To gain more insight into the effect of polymers on improving oil recovery efficiency, besides polymers with chain length *N* = 150, another two types of polymers with chain length *N* = 100 and 250 were also used as flooding agents in the oil displacement simulations. As shown in figure 3*b–d*, the displacing behaviours of oil by polymers with different chain lengths exhibit significant differences. For *N* = 100, only part of the oil droplet is pulled out of the dead end. For *N* = 150 and 250, the whole oil droplet is displaced by polymers. In particular, the injected PV needed for complete oil displacement by polymers *N* = 150 is much larger than that by polymers *N* = 250, indicating that more polymers will be required to displace the residual oil droplet. In addition, the configuration of oil droplet after polymer flooding is affected by the polymer's chain length. Hydrocarbon molecules in the oil droplet tend to gather when the chain length of polymers is longer. This is because the pulling force applied by polymers on the oil droplet is much stronger than the adhesive interaction between oil droplet and dead-end walls, resulting in hydrocarbon molecules moving together as a whole. Based on the above analyses, it can be inferred that polymers with longer chain length can effectively displace more oil using less material. It should be noted that increasing the chain length of the polymer can also bring issues that are deleterious for oil recovery in real field conditions. For example, if a polymer chain is too long, plugging and increased adsorption will occur and affect the propagation of the polymer [4]. Also, using long-chain polymer requires more polymer to be injected, which will increase the cost of oil recovery. In addition, because the properties of the oil reservoirs are highly variable, a general flooding agent for oil recovery cannot be defined [40]. Therefore, we do not suggest increasing the chain length of polymer to a very large value. The issues of plugging, increased adsorption and increased economics should be taken into consideration when choosing the appropriate polymer chain length.

**Figure 4.**
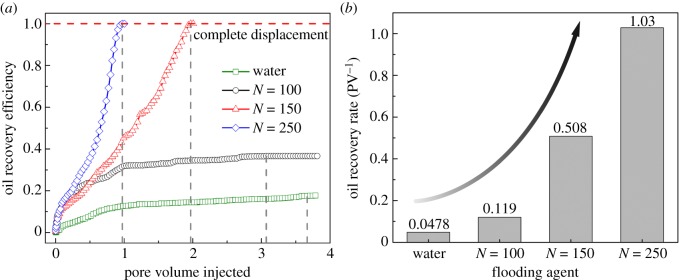
(*a*) Oil recovery efficiency during flooding process using different flooding agents. Reaching the horizontal red dashed line means the whole oil droplet is displaced out of the dead end. The vertical grey dashed lines give the corresponding injected pore volume when oil recovery efficiency reaches a steady value. (*b*) Oil recovery rate of different flooding agents.

Furthermore, oil recovery efficiencies for water flooding and polymer flooding were calculated and are plotted in figure 4*a*. Here, oil recovery efficiency is defined as the ratio of the mass of hydrocarbon molecules outside the dead end to that of the total oil droplet. As shown in figure 4*a*, as more flooding agents are injected into the nanopore, oil recovery efficiency gradually increases and finally reaches a steady value. Corresponding to the results illustrated in figure 3, polymer flooding with a longer polymer chain exhibits more effective oil displacement, while water flooding shows the lowest recovery efficiency. To directly compare the displacement ability of different flooding agents, we defined a parameter, oil recovery rate, as a quantitative evaluation standard. The oil recovery rate is given as the ratio of the final oil recovery efficiency to the injected PV needed to reach the final oil recovery efficiency, representing the oil recovery efficiency per unit injected PV during the displacement process. A higher rate indicates that more oil can be recovered using less flooding agent. Figure 4*b* illustrates the calculated oil recovery rate of different flooding agents. Clearly, polymers have better performance in oil recovery than water. For polymers, the oil recovery rate tends to be higher when chain length is longer.

### 3.2. Pulling effect mechanism

**Figure 5.**
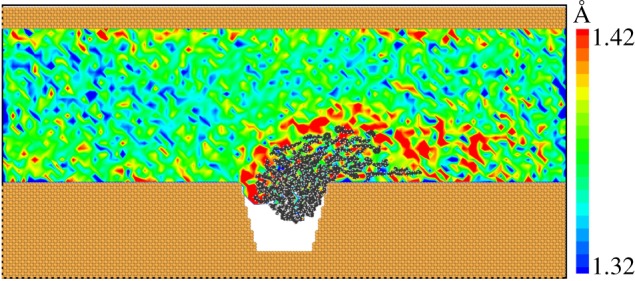
Bond length contours of polymers with chain length *N* = 250 during flooding process when the injected pore volume is 0.74. The oil droplet is also illustrated in the corresponding position.

As discussed above, polymer flooding can improve oil recovery efficiency compared with water flooding. To investigate the oil displacement mechanism of polymer flooding, we analysed the displacement processes and found that the pulling effect is a vital mechanism. Polymer has the property of viscoelasticity. On one hand, polymer flooding can increase the viscosity of the drive fluid to improve volumetric sweep efficiency macroscopically [13], which is already well known. On the other hand, polymers with a long curly chain structure were reported to possess elastic response when flowing through porous media [24]. Owing to the elastic nature of polymers, a strong pulling effect could be exerted on the residual oil droplet. As polymers are macromolecules with long chains, when flowing along the nanopore they stretch, recoil and entangle with each other [41], as shown in figure 1*a*. After coming into contact with the oil droplet, the entangled polymers surround the oil droplet under the adhesive interaction between them. As the polymers continue to flow, the forepart of the polymer chain away from the oil droplet tends to move forward, whereas the other part of the chain near the oil droplet tends to attach to the droplet. Thus, this separating tendency causes the polymer chains to stretch. As a result, pulling force is formed and applied to the oil droplet. We measured the polymer's bond length distribution during oil displacement, as shown in figure 5. A higher bond length indicates that the polymer chains are stretched and the pulling force is correspondingly formed. The average bond length of polymer in the main pore channel is about 1.37 Å. It is obvious that the bond length of polymers around the oil droplet is higher than that in the main pore channel and the length extension is over 3.65%, which means the pulling force is formed around the oil droplet. This strong evidence proves that the oil droplet is indeed displaced via pulling effect applied by polymer chains. However, for water flooding, flowing water interacts with the trapped oil droplet only by shearing, which is significantly inefficient for displacing the oil droplet. Besides shearing force, pulling force is also an important interaction between flowing polymers and the trapped oil droplet. The incomplete oil displacement of polymer flooding with a shorter polymer chain (*N* = 100) is mainly due to insufficient pulling force. When polymer solution is injected into the oil reservoir, viscoelastic polymers can not only push forward the oil adsorbed on pore walls like non-elastic fluids do, but also pull out the oil trapped in the dead ends on the surrounding walls of nanopores. As a comparison, the result presented in figure 3*a* demonstrates that water flooding is almost powerless for an oil droplet trapped in a dead end. Water molecules cannot apply a pulling effect on oil molecules to overcome the oil–wall interaction in nanoscale confinement. Thus, polymer flooding can remarkably improve microscopic oil recovery efficiency.

### 3.3. Effect of viscoelasticity

As the stretching and recoiling of polymer chains are important characteristic behaviours of a polymer's elastic property, it can be inferred that the key factor in the outstanding oil recovery performance of polymer flooding lies in the viscoelasticity of polymers, especially elasticity. Moreover, the difference in the oil displacement ability of polymers with varying chain length is caused by their differences in viscoelastic properties. So here we investigated the effect of viscoelasticity of polymers. A series of equilibrium MD simulations of pure polymers was conducted. For comparison, we also investigated the viscoelasticity of water using the same method.

As a key function of linear rheology, the stress relaxation modulus *G*(*t*) fully characterizes the linear rheology of a polymeric system with given external parameters. Based on the fluctuation–dissipation theorem, *G*(*t*) can be calculated by

3.1 $$G(t)=\frac{V}{k_{B}T}\langle \sigma_{xy}(t+\tau )\sigma_{xy}(\tau )\rangle ,$$

where *V* is the volume of the simulation box, *k*_B_ is the Boltzmann constant, *T* is the temperature and *xy* is any two orthogonal directions [42–44]. This equation averages *G*(*t*) in respect to initial time *τ*. Specifically, average in this context means the following time integral:

3.2 $$G(t)=\frac{V}{k_{B}T}\frac{1}{t_{\mathrm{sim}}-t}\int_{0}^{t_{\mathrm{sim}}-t}\sigma_{xy}(t+\tau )\sigma_{xy}(\tau )\;\text{d}\tau ,$$

where *t*_sim_ is the total simulation time. For simplicity, it is not necessary to explicitly mention *τ* and it can be omitted in the correlation functions. The above integral can be simplified as

3.3 $$G(t)=\frac{V}{k_{B}T}\langle \sigma_{xy}(t)\sigma_{xy}(0)\rangle .$$

To improve the statistical accuracy of the calculation results, we calculated the equilibrium stress auto-correlation functions and averaged over different directions because our simulation systems are isotropic. And the final expression of stress relaxation modulus *G*(*t*) is

3.4 $$\begin{array}{rl} G(t) & \;=\frac{V}{5k_{B}T}[\left\langle \sigma_{xy}(t)\sigma_{xy}(0)\rangle +\langle \sigma_{yz}(t)\sigma_{yz}(0)\rangle +\langle \sigma_{xz}(t)\sigma_{xz}(0)\right\rangle ]\\ & \;\;+\frac{V}{30k_{B}T}[\left\langle N_{xy}(t)N_{xy}(0)\rangle +\langle N_{yz}(t)N_{yz}(0)\rangle +\langle N_{xz}(t)N_{xz}(0)\right\rangle ], \end{array}$$

where *N_αβ_ = σ_αα_* − *σ_ββ_* [45].

**Figure 6.**
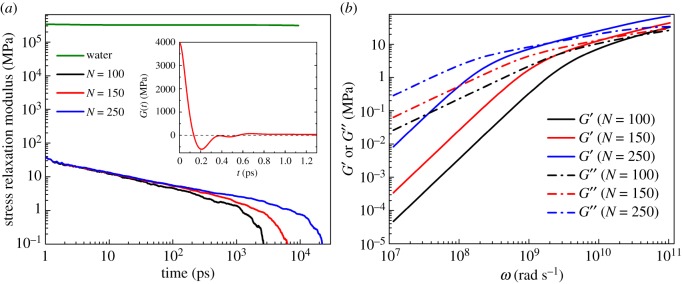
(*a*) Stress relaxation modulus *G*(*t*) for water and polymers with chain length *N* = 100, 150 and 250. The inset illustrates the same data for *N* = 100 short time-scale fluctuations at an early time arising from bond interactions. (*b*) Storage modulus *G*′(*ω*) and loss modulus *G*′′(*ω*) for different chain length polymers calculated by Maxwell modes fit to the data of *G*(*t*). Solid and dash dot lines denote the storage modulusand the loss modulus, respectively.

In calculating the stress auto-correlation functions, for each time *t*, a running average was performed between 0.9 *t* and 1.1 *t* to reduce the noise of the signal [43,46]. The corresponding *G*(*t*) curves of water and polymers with different chain lengths were plotted in figure 6*a*. For water, the *G*(*t*) curve is a horizontal line, which means water does not manifest the behaviour of stress relaxation. This proves water is not a viscoelastic fluid. For polymers, a clear non-Newtonian behaviour of stress relaxation can be observed, which means that stress gradually decreases in response to the same amount of strain over many orders of magnitude in time. The short-time oscillation of *G*(*t*) shown in the inset is mainly caused by the rapid fluctuations of the stiff bond interactions. As expected, the intermediate time scale behaviour of the stress auto-correlation function can be quantitatively described using the Rouse model *G*(*t*) ∝ *t*^−1/2^. Also, it can be seen that the terminal relaxation time increases as chain length increases.

With stress relaxation modulus *G*(*t*) obtained, the frequency-dependent modulus of polymers can be generated, namely the storage modulus *G*′(*ω*) and the loss modulus *G*′′(*ω*). The theoretical relationship between the moduli and the stress relaxation modulus is

3.5 $$G^{*}(\omega )=G^{\prime }(\omega )+\text{i}G^{\prime \prime }(\omega ),$$

where G* is the complex modulus and i is the imaginary number.

In practical application, by recording the in-phase and out-of-phase response of polymers under oscillatory conditions, one can measure the sine and cosine Fourier transforms of *G*(*t*), which are the so-called storage and loss moduli. Here, a simple way was used to calculate *G*′(*ω*) and *G*′′(*ω*). We firstly used RepTate toolbox [47] to fit *G*(*t*) with a sum of exponentials:

3.6 $$G(t)=\sum_{i=1}^{m}G_{i}\exp\left(\frac{-t}{\tau_{i}}\right),$$

where *G_i_* and *τ_i_* are the amplitude and relaxation time of the Maxwell mode *i*, and *m* is the number of Maxwell modes summed. Then *G*(*t*) can be easily Fourier transformed to *G*′(*ω*) and *G*′′(*ω*) through the equations below [43]:

3.7 $$G^{\prime }(\omega )=\sum_{i=0}^{m}G_{i}\frac{{\left(\omega \tau_{i}\right)}^{2}}{1+{\left(\omega \tau_{i}\right)}^{2}}\text{ and }G^{\prime \prime }(\omega )=\sum_{i=0}^{m}G_{i}\frac{\omega \tau_{i}}{1+{\left(\omega \tau_{i}\right)}^{2}}.$$

The results of the above transformations are plotted in figure 6*b*. Storage modulus represents the stored energy caused by elastic deformation, reflecting the elastic properties of polymers. Loss modulus represents the energy dissipated as heat during deformation, reflecting the viscous properties of polymers. *G*′(*ω*) and *G*′′(*ω*) curves show that with the increase of chain length, both storage modulus and loss modulus increase, indicating that both elasticity and viscosity are strengthened during polymer flooding with longer polymer chain lengths. In addition, the inverse of the frequency at the cross-over point of *G*′(*ω*) and *G*′′(*ω*) curves gives the relaxation time of polymers. As expected, relaxation time increases with the increase of polymer chain length. Relaxation time is also a characteristic parameter representing the elastic behaviour of polymers. Longer relaxation time implies that more time is needed for polymer chains to adjust their configuration. It can be inferred that polymers with longer chain length have stronger elasticity and tend to elongate more when flowing along pore throats, thus enabling them to reach deeper in the pores and apply stronger pulling force on the trapped oil droplet and improve microscopic oil recovery efficiency.

## 4. Conclusion

In this study, we theoretically investigated the molecular mechanism of viscoelastic polymer EOR in nanopores. By conducting MD simulations, the dynamic process of trapped oil droplet displacement from a dead end within a nanopore was studied. We demonstrated that the key factor of the excellent performance of polymer flooding in oil recovery lies in the unique elastic property of polymer molecules. The pulling effect mechanism was confirmed to be the main contribution to the enhanced microscopic oil displacement of polymer flooding compared with simple water flooding. We also found that polymers with longer chain length tend to displace more trapped oil with less injected PV, resulting in higher microscopic oil recovery efficiency. The analyses of viscoelasticity of polymers revealed that both storage modulus and relaxation time become higher when polymer chain length is longer, strengthening the effect of polymer elasticity. This enhancement of elasticity enables polymers to stretch more in the pore and apply stronger pulling force on the oil droplet trapped in dead ends, which is beneficial for improving oil displacement efficiency. Our work confirms improvement of oil recovery efficiency by polymer flooding in nanoscale confinement. These results and findings contribute to understanding the microscopic oil displacement mechanisms of polymer EOR and may provide guidance for practical oil recovery processes.

## Data accessibility

The data and code for the analyses of simulation results are available at the Dryad Digital Repository (https://doi.org/10.5061/dryad.61g8620) [48].
